# Economic burden of influenza-associated hospitalizations and outpatient visits in Bangladesh during 2010

**DOI:** 10.1111/irv.12254

**Published:** 2014-04-22

**Authors:** Mejbah U Bhuiyan, Stephen P Luby, Nadia I Alamgir, Nusrat Homaira, Abdullah A Mamun, Jahangir A M Khan, Jaynal Abedin, Katharine Sturm-Ramirez, Emily S Gurley, Rashid U Zaman, ASM Alamgir, Mahmudur Rahman, Marc-Alain Widdowson, Eduardo Azziz-Baumgartner

**Affiliations:** a icddr,b, Centre for Communicable DiseasesDhaka, Bangladesh; b Centers for Disease Control and PreventionAtlanta, GA, USA; c James P. Grant School of Public Health, BRAC UniversityDhaka, Bangladesh; d Oxford Policy ManagementOxford, UK; e Institute of Epidemiology, Disease Control and ResearchDhaka, Bangladesh

**Keywords:** Bangladesh, cost, hospitalization, influenza, outpatient

## Abstract

**Objective:**

Understanding the costs of influenza-associated illness in Bangladesh may help health authorities assess the cost-effectiveness of influenza prevention programs. We estimated the annual economic burden of influenza-associated hospitalizations and outpatient visits in Bangladesh.

**Design:**

From May through October 2010, investigators identified both outpatients and inpatients at four tertiary hospitals with laboratory-confirmed influenza infection through rRT-PCR. Research assistants visited case-patients' homes within 30 days of hospital visit/discharge and administered a structured questionnaire to capture direct medical costs (physician consultation, hospital bed, medicines and diagnostic tests), direct non-medical costs (food, lodging and travel) and indirect costs (case-patients' and caregivers' lost income). We used WHO-Choice estimates for routine healthcare service costs. We added direct, indirect and healthcare service costs to calculate cost-per-episode. We used median cost-per-episode, published influenza-associated outpatient and hospitalization rates and Bangladesh census data to estimate the annual economic burden of influenza-associated illnesses in 2010.

**Results:**

We interviewed 132 outpatients and 41 hospitalized patients. The median cost of an influenza-associated outpatient visit was US$4.80 (IQR = 2.93–8.11) and an influenza-associated hospitalization was US$82.20 (IQR = 59.96–121.56). We estimated that influenza-associated outpatient visits resulted in US$108 million (95% CI: 76–147) in direct costs and US$59 million (95% CI: 37–91) in indirect costs; influenza-associated hospitalizations resulted in US$1.4 million (95% CI: 0.4–2.6) in direct costs and US$0.4 million (95% CI: 0.1–0.8) in indirect costs in 2010.

**Conclusions:**

In Bangladesh, influenza-associated illnesses caused an estimated US$169 million in economic loss in 2010, largely driven by frequent but low-cost outpatient visits.

## Introduction

Each year approximately 5% of adults and 20% of children worldwide develop an influenza infection.^1^–^3^ In Bangladesh, influenza virus is an important contributor to the acute respiratory illness burden.^4^–^6^ Influenza-associated illnesses result in direct medical costs, including costs for consultation, medications, hospitalization, and laboratory tests. In 2003, the estimated direct medical cost for annual influenza illness was US$10·4 billion in the U.S.^7^ Influenza illnesses also result in indirect losses related to school and workplace absenteeism.^8^,^9^ The projected lost earning because of influenza illness was US$16·3 billion in the U.S. during 2003.^7^ In France and Germany, the estimated loss of productivity ranged between US$10 - 15 billion per year.^10^ In Thailand, influenza illnesses caused a US$23–63 million economic loss in 2003–2004.^11^

In Bangladesh, two studies have estimated the cost of acute respiratory illnesses. In 2007, a study conducted in the largest pediatric hospital in Bangladesh estimated that the mean medical cost of hospitalization per episode of pneumonia among children aged <5 years was US$95, which was >50% of the monthly income of 75% of families studied.^12^ In 2009, a study in an urban neighborhood of Dhaka estimated that the median ambulatory medical cost of an episode of influenza-like illness was US$6, which represented 9% of the monthly household expenditure of respondents.^13^ Although neither of the previous cost-of-illness studies was conducted among patients with laboratory-confirmed influenza, these data suggest that the cost of influenza-associated illnesses may be expensive for affected families in Bangladesh because the majority of healthcare costs are paid out-of-pocket with the yearly per capita income was only US$ 770 in 2011.^12^–^17^

Influenza burden can be reduced by pharmaceutical (e.g., vaccination) and non-pharmaceutical (e.g., hand and respiratory hygiene, social distancing) interventions.^18^–^20^ Routine influenza vaccination is the most effective way to prevent and control influenza illness, and at US$4–$25, a dose is generally considered a cost-effective intervention in middle- and high-income countries for certain risk groups.^21^–^23^ The value of influenza preventive investments remains unknown in low-income countries including Bangladesh because information on the costs associated with influenza illness is limited. Understanding the costs of influenza-associated illness in Bangladesh may help health authorities assess the cost-effectiveness of influenza prevention programs. We conducted a study to estimate the cost-per-episode of laboratory-confirmed influenza illness and used previously published influenza incidence data^4^,^5^ to estimate the annual economic burden of influenza-associated illness in Bangladesh in 2010.

## Methods

### Study settings

**Figure 1:**
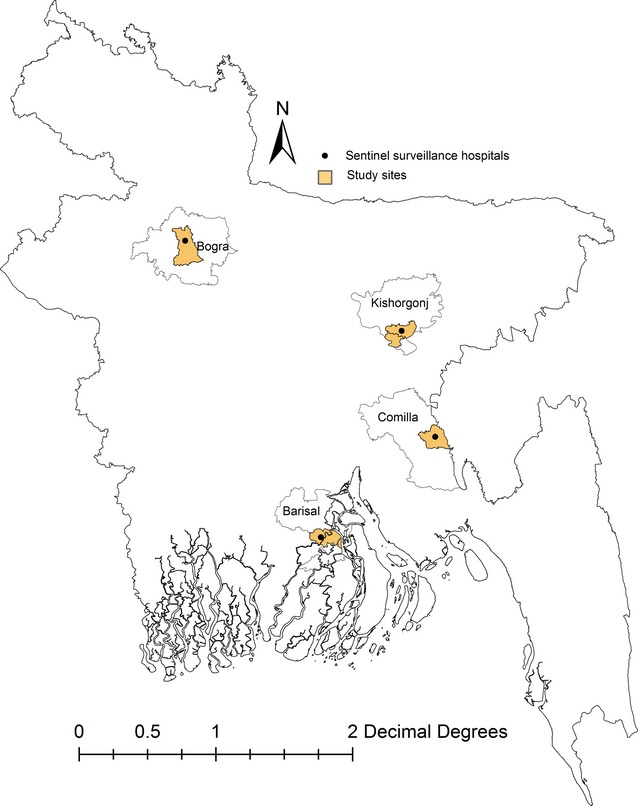
Sentinel influenza surveillance catchment sites in Bangladesh, 2010.

The Institute of Epidemiology, Disease Control and Research (IEDCR), the Government of Bangladesh and icddr,b has been conducting a sentinel influenza surveillance program at one private and three government tertiary hospitals in four districts of Bangladesh, Kishorgonj, Comilla, Bogra, and Barisal since 2008^4^ (Figure1). We conducted this study among the population resident in the catchment areas of these surveillance sites.

### Study population

In Bangladesh, influenza activity typically peaks during May through October.^6^ During May through October, 2010, investigators identified inpatients and outpatients from the influenza surveillance program database with laboratory-confirmed influenza disease, defined them as case-patients, and collected the contact information of each case-patient. Research assistants telephoned each case-patient or household members to obtain permission for a home visit and then visited case-patient's homes within 30 days of their outpatient visit or hospital discharge. If the case-patient or a household member was unavailable during the first home visit, research assistants made two more visits to the household to contact them.

### Data collection

Research assistants interviewed the person of the case-patient's family who knew about the illness episode and the associated costs. Research assistants used a structured questionnaire to obtain socioeconomic information, health-seeking behavior of the case-patient, expense information associated with the illness episode (capturing both direct and indirect costs), and information on any coping strategies used by the household to pay for the treatment.

#### Direct cost components

We categorized direct cost items into medical and non-medical items.^24^ Medical cost items included hospital registration fees, bed rental, medications, laboratory tests, and informal payments^a^ (if any) incurred during visits to the hospital. Research assistants used prescription or discharge reports to record all the medications and laboratory tests^b^ that case-patients obtained during the illness episode. Case-patients or their household members distinguished which medications and laboratory tests they received from the hospital free-of-charge (hospital subsidized cost) and which they purchased (out-of-pocket). We used hospital receipts to record registration fees and bed rental. Research assistants also collected self-reported illness-related non-medical cost items such as food, caregiver's lodging, and transportation costs from patients or their family members. To calculate routine healthcare costs, we used WHO-Choice estimates for healthcare service costs per bed-day (for hospitalized patients) or per outpatient visit at tertiary level hospitals in Bangladesh which included personnel (healthcare providers and support staff salary), capital, and patients food costs but excluded the cost of drugs and diagnostic tests.^25^

#### Indirect cost components

We recorded self-reported productivity loss for case-patients and their caregivers during the illness. For employed case-patients and caregivers, we recorded the number of work days lost because of illness or while caring for sick family members. We did not count weekends or official holidays. For daily-wage earners or homemakers, any day lost because of illness or while caring for sick family members was considered a lost work day. We did not count values for days lost on reduced activity days (for example, half-day of work), for school absenteeism, or for influenza-associated deaths.

### Data analysis

#### Cost-per-episode of influenza-associated illness

We calculated all medicines and laboratory costs using price lists from local drug stores and laboratories. We estimated out-of-pocket costs and hospital subsidies (hospital supported cost) for medicine and laboratory tests. We added medical and non-medical costs to estimate the total direct cost-per-episode. We multiplied hospitalization days by an estimated cost per bed-day and cost per outpatient visit to obtain the healthcare service cost.

We used the human capital approach to calculate the productivity loss for employed and daily-wage earner case-patients and caregivers.^26^ We collected self-reported daily wage rates from case-patients and caregivers and assigned a monetary loss by multiplying missed work days by daily wages. In Bangladesh, female family members were commonly homemakers and provide the majority of hands on care to ill family members in hospital and at home.^27^,^28^ Although this group of caregivers are mostly unemployed, we attempted to calculate their productivity loss using the minimum wage rate set by the Government of Bangladesh during 2010 which was 100 taka/day (US$ 1·4/day)^29^ and multiplied this rate by the number of days they cared for the case-patient. We summed the productivity loss of each case-patient and caregiver to estimate the total indirect cost per illness for each case-patient. We did not attribute a cost to productivity loss when a case-patient or their caregiver reported that the illness episode did not disrupt or caused only a modest disruption in their usual activities.

We added the direct and indirect costs of outpatient visits to obtain the total cost-per-episode of influenza-associated outpatient visits. Similarly, we obtained the total cost-per-episode of an influenza-associated hospitalization. We converted our cost estimates from Bangladeshi currency (taka) to US dollars using an average exchange rate (US$ 1 = 70 taka)^30^ for the data collection period May to October 2010. We estimated means (range or 95% CI) if the data were normally distributed or medians (interquartile range, IQR) otherwise.

#### Annual economic burden of influenza-associated illness

We applied the published incidence rate for influenza hospitalizations among patients aged <5 years^5^ and patients aged ≥5 years in 2010^4^ to the national census population (150 433 428) in 2010^31^ to estimate the number of hospital admissions attributable to influenza. We multiplied the median cost-per-episode for influenza hospitalization by the estimated number of hospitalizations. Similarly, we estimated the number of influenza cases managed in outpatient settings nationwide using data from 2010 (17/100 person-years).^4^ This figure was multiplied by the cost-per-episode for an outpatient visit. We used a Monte Carlo simulation to resample our cost components 1000 times to generate a 95% CI of the estimates.^7^,^32^

#### Principle component analysis

We performed principal component analysis on household income and assets to construct an wealth index and divided the households into five quintiles according to the principal component score.^33^

### Ethical consideration

We obtained written informed consent from case-patients aged ≥18 years, from parents of case-patients that were aged <18 years, and verbal assent from participants aged 7–17 years. The study protocol was approved by icddr,b and CDC's institutional review committee.

## Result

### Characteristics of study respondents

During May through October 2010, we interviewed 173 case-patients with laboratory-confirmed influenza (Table1). Nineteen (14%) of 132 outpatients and 15 (37%) of 41 hospitalized patients were earning members of the household. Fifty-five percent (73/132) of outpatients and 73% (30/41) of hospitalized patients visited other healthcare providers before visiting the surveillance hospitals.

**Table 1:** Characteristics of study respondents with influenza-associated illnesses in Bangladesh, May–October, 2010

Characteristics	Outpatients	Hospitalized patients	*P* value*
	*N* = 132	*N* = 41	
Enrolled from a public hospital, *n* (%)	111 (84)	25 (61)	
Enrolled from a private hospital, *n* (%)	21 (16)	16 (39)	
Male, *n* (%)	69 (52)	23 (56)	0·6
Age distribution (years), *n* (%)			
0–4	49 (37)	5 (12)	0·002
5–19	44 (33)	12 (29)	0·6
20–49	35 (27)	10 (24)	0·7
50+	4 (3)	14 (34)	<0·001
Household monthly income (US$), median (IQR)	129 (86–176)	136 (100–186)	0·7
Number of household members, median (IQR)	5 (4–6)	5 (4–7)	0·8
Days between symptom onset to hospital visit, mean (range)	3 (2–5)	3 (2–5)	0·8
Hospital stay (days), median (IQR)	N/A	3 (3–4)	–

* Comparisons are between outpatients and hospitalized patients.

### Costs for influenza-associated illness

Table2 summarizes the costs for influenza-associated illness in Bangladesh.

**Table 2:** Direct, indirect, and total cost-per-episode of influenza-associated illnesses in four hospitals in Bangladesh in 2010

Parameter	Hospitalized patient			Outpatient		
	
	*n*	Median	IQR	*n*	Median	IQR
Direct cost (US$)						
Medication cost	41	18·20	11·02–28·57	132	2·30	1·30–4·17
Diagnostic cost	34	12·21	6·42–19·00	6	7·28	3·57–8·42
Transportation cost*	41	4·00	2·00–8·71	124	0·57	0·28–1·14
Food cost	36	3·14	1·55–5·65	5	0·35	0·32–0·42
Hospital bed charge	17	6·95	4·65–6·98	N/A		
Informal payment	14	0·64	0·28–0·85	0	0	0
Healthcare service cost	41	13·53	13·53–18·04	132	0·94	0·94–0·94
Total direct cost/episode	41	59·75	40·43–82·71	132	4·21	2·91–6·53
Indirect cost (US$)						
Productivity loss of case-patient	20**	21·42	11·07–45·24	22***	11·42	4·28–21·42
Productivity loss of caregiver (s)	38	10·00	5·71–17·14	7	2·14	1·13–3·57
Total indirect cost/episode	40	22·25	10·00–35·24	27	9·10	2·82–17·14
Total cost/episode	41	82·20	59·96–121·56	132	4·80	2·93–8·11

* Transportation costs included round trip expense for case-patient from home to hospital and caregivers travel expense for hospital visits.

** Includes five unemployed homemakers.

*** Includes eight unemployed homemakers.

#### Influenza-associated outpatient visits

The median direct cost for influenza-associated outpatient clinic visits was US$3·66 (IQR = 2·79–5·92) at public hospitals and US$7·08 (IQR = 6·22–12·22) at the private hospital (*P* < 0·001). Eighty-six (77%) of 111 case-patients who visited the public hospital clinics received at least one subsidized medication and/or laboratory test and the subsidy was 34% (median) of the total cost of medications and laboratory tests, whereas one (5%) of 21 case-patients who visited the private hospital received subsidized medications and laboratory tests and the subsidy was 100%. In total, 26 (23%) case-patients at the public hospitals and 20 (95%) case-patients at the private hospital paid 100% of their medication and laboratory test costs out-of-pocket. The cost of medications was 75% (median, IQR = 58–86%) of the total direct outpatient cost.

Fourteen (74%) of 19 income earning case-patients missed a median of eight work days (IQR = 3–15) at a median loss of US$2·26 (IQR = 0·95–2·85) per day because of illness. Homemakers were the primary caregivers for ambulatory case-patients, but all reported carrying out their usual activities without interruption or with modest disruption while caring for ill family members, so no value was attributed to these events. Other caregivers such as fathers, husbands, and grandparents (*n* = 7) missed a median of one work day (IQR = 1–3) at a median cost of US$1·42 (IQR = 0·47–2·14) per day.

The median cost-per-episode for influenza-associated outpatient visits was US$4·80 (IQR = 2·93–8·11) for all patients, US$4·22 (IQR = 2·79–7·04) for patients in public hospitals, and US$8·59 (IQR = 6·22–14·36) in the private hospital (Table2). The out-of-pocket cost was 2% (median, IQR: 1·0–3·7) of the monthly income for those families.

We multiplied the incidence rate influenza at outpatient settings (17/100 person-year)^4^ and national census population in 2010 and estimated that 25 573 683 persons sought care for influenza illness at outpatient settings throughout Bangladesh. Multiplying the estimated annual influenza cases at outpatient clinics by the median cost per influenza-associated outpatient visits (US$4·80), we estimated that the direct cost of influenza-associated outpatient visits was US$108 million throughout the country (95% CI: 76–147) (Table3). Influenza-associated outpatient visits ranged between an estimated 52 329–88 767 years of work loss and represented US$59 million (95% CI: 37–91) in indirect losses.

**Table 3:** Estimated annual cost of influenza-associated illnesses (in US$ millions) in Bangladesh in 2010

Parameter	Hospitalized patient		Outpatient

	<5 years	≥5 years	
	Cost (95% CI)	Cost (95% CI)	Cost (95% CI)
Total medication and diagnostic test costs	0·18 (0·02–0·41)	0·52 (0·18–0·98)	59·25 (42·21–80·83)
Hospital subsidized costs	0·01 (0·001–0·02)	0·001 (0·004–0·02)	7·30 (5·58–9·45)
Out-of-pocket costs	0·14 (0·02–0·30)	0·51 (0·18–0·98)	43·84 (30·79–66·19)
Total transportation* costs	0·02 (0·002–0·06)	0·07 (0·02–0·14)	13·18 (8·38–18·91)
Other costs**	0·01 (0·001–0·03)	0. 11 (0·04–0·21)	3·98 (2·79–5·20)
Healthcare service costs	0·15 (0·02–0·34)	0·23 (0·09–0·39)	24·03 (18·3–31·1)
Total direct costs	0·37 (0·05–0·83)	1·05 (0·39–1·90)	107·65 (76·06–147·17)
Total indirect costs	0·04 (0·006–0·10)	0·38 (0·14–0·68)	59·02 (37·48–91·03)

* Transportation costs included round trip expense for case-patient from home to hospital and caregivers travel expense for hospital visits.

** Other costs included expenses for hospital registration, informal payments, food, hospital bed rental, and caregiver lodging.

#### Influenza-associated hospitalization

The median direct cost of influenza-associated hospitalizations was US$54·63 (IQR = 28·78–68·78) in public hospitals and US$76·26 (IQR = 61·90–101·87) in the private hospital (*P* < 0·001). Eleven (44%) of 25 case-patients in the public hospitals received at least one subsidized medication and/or laboratory tests and the subsidy was 5% (median) of total cost of medication and laboratory tests, whereas two (13%) of 16 case-patients in the private hospital received subsidized medication and/or laboratory tests and the subsidy was 29% of the total cost of medication and laboratory tests. In total, 14 (56%) case-patients at the public hospitals and 14 (87%) case-patients at the private hospital paid 100% of medication and laboratory costs out-of-pocket. The medication cost was 46% (median, IQR = 35–56%) of the total direct cost (Table2).

Eleven case-patients missed a median of 10 school days (IQR = 7–17) because of illness. Fifteen (100%) of 15 income earning case-patients missed a median of 13 productive days (IQR = 6–20) at a median of US$2·37 (IQR = 1·42–5·01) per day because of illness. Thirty-eight caregivers, including 34 homemakers, missed a median of seven productive days (IQR = 4–12) at a median of US$1·42 (IQR = 1·42–1·42) per day.

The median cost-per-episode was US$82·20 (IQR = 59·96–121·56) for all influenza-associated hospitalizations, US$67·25 (IQR = 48·37–86·14) in public hospitals, and US$103·41 (IQR = 83·20–145·54) in the private hospital (Table2). The out-of-pocket cost was 28% (median, IQR: 16–45) of the monthly income for affected families.

We estimated that 13 553 cases aged <5 years and 17 039 cases aged ≥5 years were hospitalized for laboratory-confirmed influenza during 2010. We estimated that in 2010, the direct costs related to influenza-associated hospitalizations in Bangladesh was US$1·4 million (95% CI: 0·4–2·6) for all ages (Table3). Similarly, influenza-associated hospitalization ranged between 288 - 1967 years of work loss and represented US$0·4 million (95% CI: 0·1–0·8) in indirect losses.

### Financial burden associated with hospitalization

Twenty-six (63%) of 41 families reported reducing their monthly food expenditure during the month of illness. In order to pay for the treatment, 25 (61%) of 41 hospitalized case-patient families borrowed money (Table4). Of these 25 families, 13 (52%) obtained loans from local community leaders at a median annual interest of 120% (IQR = 15–120%); the remaining 12 families obtained interest-free loans from relatives.

**Table 4:** Out-of-pocket cost to monthly household income and the coping strategies that families used to meet treatment cost for influenza-associated hospitalization, by income quintiles, Bangladesh, May-October 2010

Parameter	Asset quintiles				

	Q1 (*n* = 8)* poorest	Q2 (*n* = 7)	Q3 (*n* = 9)	Q4 (*n* = 9)	Q5 (*n* = 8) richest
Household monthly income, median (IQR)	91 (61–125)	114 (81–143)	124 (109–171)	147 (143–336)	260 (182–311)
Out-of-pocket costs** as percentage of their monthly income, median (IQR)	37 (27–49)	36 (21–54)	16 (14–26)	34 (22–58)	26 (11–29)
Coping strategy***					
Used savings	1 (13)	0	0	2 (22)	0
Received contribution from relatives	1 (13)	2 (29)	1 (11)	0	1 (13)
Borrowed money	8 (100)	6 (86)	6 (67)	5 (56)	0

Data are frequency (%) or otherwise mentioned.

* *n* indicates the number of families in the asset quintile.

** Includes cost for medicine, diagnostic tests, transportation, hospital registration, informal payments, food, hospital bed rental, and caregiver lodging; does not include productivity loss associated with illness.

*** Multiple-choice-type questions.

## Discussion

Our study suggests that the annual economic burden of influenza in Bangladesh was US$ 169 million in 2010. Our estimates of the median cost-per-episode of influenza-associated hospitalization (US$82) and influenza-associated outpatient visits (US$ 4·8) were consistent with other cost estimates of respiratory illness in Bangladesh, US$ 6·2 for ILI^13^ and US$ 82 for pneumonia.^12^ This study highlights that the annual national costs for ambulatory influenza-associated illness are greater than influenza-associated hospitalization costs, which is consistent with previous studies in the U.S.^7^,^34^ and Thailand.^11^ Although influenza-associated outpatient visit costs in Bangladesh were one-tenth of the hospitalization cost-per-episode for influenza, the greater frequency of outpatient visits compared with hospitalizations results in a substantial annual economic burden. In contrasts with previously published data from the U.S.^7^ and Thailand,^11^ our indirect cost estimates was lower compared with direct costs, probably because of the comparatively low per capita annual income in Bangladesh (US$ 770) compared with the U.S.(US$ 48 620) and Thailand (US$ 4400).^17^ Our cost estimates were, indeed, lower than estimates from neighboring middle- or other high-income countries^7^,^11^,^34^ because the healthcare system is radically different. The diagnostic tests and routine procedures common in middle- and high-income countries for patients with ILI are infrequently practiced in Bangladesh.^35^ We found that few case-patients with ILI at outpatient setting were sent for diagnostic tests, probably because physicians perceived case-patient's illness as mild and likely self-limited.

We estimated that influenza-associated hospitalizations comprised 4% of the total annual economic burden of influenza illness. However, the amount incurred for an episode of influenza-associated hospitalization frequently resulted in financial hardship to affected families, particularly the poorest families, because the medical costs were typically paid out-of-pocket. Similar to other low-income countries, there is a no public health insurance to cover healthcare costs in Bangladesh.^16^,^36^ A review on healthcare payment in low-and middle-income countries in Asia illustrated that heavy reliance on out-of-pocket expenditure for healthcare often had an immediate effect on household living standard such as reduction in expenses on necessary household items including food and education for children.^37^ In this study, the direct consequence of hospitalization expenses was reducing monthly food expenditures. These data highlight an indirect impact of influenza illness on household nutritional status, which is concerning given that 41% of children aged <5 years suffer from malnutrition in Bangladesh.^38^ Borrowing money was the primary coping strategy to pay for the cost of treatment for all families except the richest families. Some families borrowed money from local money lenders at a very high interest. Similar coping strategies are documented in other resource poor settings.^12^,^39^ In low-income countries like Bangladesh, where nearly half the population lives below the national poverty line,^40^ borrowing money to meet hospitalization costs could put affected families at risk for long-term debts, contributing to the cycle of poverty.^37^,^39^ Research to explore alternative ways of healthcare financing may be useful in this and similar settings to lessen the impact of hospitalization for influenza on households.

Our study has some important limitations. We believe our influenza-associated economic burden estimates are conservative for several reasons. Additional costs that respondents incurred while visiting other healthcare providers prior to visiting sentinel hospitals was not included in our analyses because of a lack of documentation of medical prescriptions hindered accurately quantifying those costs. We estimated the economic burden of influenza-associated respiratory illness only and therefore likely excluded the associated costs for complications following influenza illnesses such as myocardial infarction and death from cardiovascular disease.^41^ We did not consider indirect cost of days with reduced activity because we assumed that the inclusion of those days would risk overreporting productivity losses. We attempted to minimize under- or overreporting cost data, by collecting expenditure data through prescription and discharge reports to substantiate cost histories. Our annual economic burden estimation was based on data from four geographically diverse hospitals and one influenza season (2010). Although the sites did not fully represent urban areas which account for 25% of country's population and are more populated than rural areas, the rate that we used was similar to rates from an urban community-based surveillance in Bangladesh (10/100 person-year) and from other settings in this region in other years.^42^–^44^ Last, our costs were of the general population and we were not able to stratify costs by risk groups for severe influenza infection such as older age or underlying comorbidity.

In Bangladesh, influenza-associated illnesses cost an estimated US$169 million during 2010. These annual costs were largely driven by the frequent but low-cost outpatient visits. The substantial lower annual cost of influenza-associated illnesses in Bangladesh compared with other settings where vaccination has been a cost-effective strategy has profound implications for the economics of routine universal influenza vaccination in Bangladesh. This study warrants investigation of cost of influenza illness among groups at high risk of complications such as healthcare workers, pregnant women, young children, the elderly, and those with chronic diseases, in order to better determine the potential value of targeted influenza vaccination programs in Bangladesh.

## Footnotes

a Informal payments included monies paid to hospital support staff for arranging beds for admitted patients on the floor of the ward, or obtaining oxygen cylinders for patients.

b Laboratory test did not include influenza testing as the test was covered by sentinel surveillance.
